# Linkage mapping bovine EST-based SNP

**DOI:** 10.1186/1471-2164-6-74

**Published:** 2005-05-19

**Authors:** Warren M Snelling, Eduardo Casas, Roger T Stone, John W Keele, Gregory P Harhay, Gary L Bennett, Timothy PL Smith

**Affiliations:** 1 US Meat Animal Research Center, Agricultural Research Service, US Department of Agriculture, Spur 18D, Clay Center, Nebraska 68933-0166, USA

## Abstract

**Background:**

Existing linkage maps of the bovine genome primarily contain anonymous microsatellite markers. These maps have proved valuable for mapping quantitative trait loci (QTL) to broad regions of the genome, but more closely spaced markers are needed to fine-map QTL, and markers associated with genes and annotated sequence are needed to identify genes and sequence variation that may explain QTL.

**Results:**

Bovine expressed sequence tag (EST) and bacterial artificial chromosome (BAC)sequence data were used to develop 918 single nucleotide polymorphism (SNP) markers to map genes on the bovine linkage map. DNA of sires from the MARC reference population was used to detect SNPs, and progeny and mates of heterozygous sires were genotyped. Chromosome assignments for 861 SNPs were determined by twopoint analysis, and positions for 735 SNPs were established by multipoint analyses. Linkage maps of bovine autosomes with these SNPs represent 4585 markers in 2475 positions spanning 3058 cM . Markers include 3612 microsatellites, 913 SNPs and 60 other markers. Mean separation between marker positions is 1.2 cM. New SNP markers appear in 511 positions, with mean separation of 4.7 cM. Multi-allelic markers, mostly microsatellites, had a mean (maximum) of 216 (366) informative meioses, and a mean 3-lod confidence interval of 3.6 cM Bi-allelic markers, including SNP and other marker types, had a mean (maximum) of 55 (191) informative meioses, and were placed within a mean 8.5 cM 3-lod confidence interval. Homologous human sequences were identified for 1159 markers, including 582 newly developed and mapped SNP.

**Conclusion:**

Addition of these EST- and BAC-based SNPs to the bovine linkage map not only increases marker density, but provides connections to gene-rich physical maps, including annotated human sequence. The map provides a resource for fine-mapping quantitative trait loci and identification of positional candidate genes, and can be integrated with other data to guide and refine assembly of bovine genome sequence. Even after the bovine genome is completely sequenced, the map will continue to be a useful tool to link observable phenotypes and animal genotypes to underlying genes and molecular mechanisms influencing economically important beef and dairy traits.

## Background

Quantitative trait loci (QTL) have been described for a number of economically important traits in cattle [1,2]. Previous genetic maps [3,4] were sufficient to map QTL to broad regions. Narrowing those regions to fine-map the QTL, and eventually identify specific genes affecting a trait requires denser genetic maps with markers that can be associated with genes. Recently, 2277 microsatellite markers were added to the bovine genetic map [5] jointly developed by the Shirakawa Institute of Animal Genetics (SIAG) and the United States Meat Animal Research Center (MARC), reducing the average interval between markers from 3.0 cM to 1.4 cM. The updated map provides a tool to refine QTL locations, but, because it predominately represents anonymous markers, provides limited information about genes underlying the QTL. A second-generation radiation hybrid (RH) map of the bovine genome, representing 1564 gene markers (1463 with human homologs) and 349 microsatellite markers that have also been placed on linkage maps has also been described [6]. The utility of this gene-rich physical map is limited, however, by relatively sparse connections to genetic markers that can be associated with animal performance. Dense genetic and physical maps (ultimately fully annotated genomic DNA sequence) are needed to efficiently identify genes, and sequence variation, responsible for phenotypic variation. Dense connections between the physical maps and genetic marker maps are also needed, to associate animal phenotypes with the underlying genes and genomic sequence.

Recent efforts have added some gene-specific markers to bovine genetic maps. Several single-nucleotide polymorphism (SNP) markers have been developed to map specific targeted genes [7,8], or positional candidate genes near QTL [9,10]. Seventy SNP markers, developed using randomly selected bovine EST with human orthologs, were added to the bovine linkage map via twopoint linkage [11]. Two-point linkages for thirty other EST-based SNPs, selected to refine the comparison of bovine chromosome (BTA) 19 with human chromosome 17, were also obtained [11]. The present study extends EST-based SNP mapping to develop a linkage map representing nearly 1000 SNPs throughout the bovine genome. This map will provide a resource for gene-based genome-wide QTL scans and fine-mapping QTL. Only a few of the SNPs added to the linkage map are likely to represent genes directly influencing production. These SNP markers will, however, further define comparative relationships between the bovine linkage map and the well-annotated human and model organism genome sequences. These EST-based SNPs may facilitate identification of positional candidate genes that could ultimately affect costs of production and consumer acceptability of meat and milk products.

## Results

Cattle genotyped for the SIAG-MARC linkage map represent four-breed crosses and backcross families [12]. F_1 _dams were produced by mating Piedmontese, Longhorn or Nelore bulls to non-inbred Hereford and Angus dams. The F_1 _dams were mated to two paternal half-sib Gelbvieh × Simmental bulls, producing full-sib four-breed cross calves by multiple ovulation embryo transfer (MOET). The backcross families were produced by mating a Nelore × Hereford bull to non-inbred Hereford dams, and a Brahman × Angus bull to non-inbred Angus dams, with full-sib backcross calves also produced by MOET. This population allows a potential of 412 informative meioses for autosomal markers [4].

The current SIAG-MARC bovine linkage maps represent 4779 markers, with 4585 markers on autosomes (Additional file 1). All of the 913 SNPs on the linkage maps (Table 1) are assigned to autosomes, including the 735 mapped in this work. In addition to the newly developed and mapped SNPs, 100 other markers including 76 previously described with two-point positions [11] were added to the multipoint autosomal linkage maps.

**Table 1:** Markers represented on SIAG-MARC bovine autosome linkage maps.

	**Informative Meioses^a,b^**			
**Marker type**	**N**	**Mean**	**Minimum**	**Maximum**
microsatellite	3612	207	11	366

single nucleotide polymorphism				
new SNP^c^	735	48	10	149
other SNP^d^	178	52	13	200

other^e^	60	69	16	307

All types	4585	174	10	366

^a^Total informative meioses.

^b ^412 potential informative meioses from bovine reference population.

^c ^newly developed and mapped EST- and BAC-based SNP.

^d ^SNP marker data contributed by other projects.

^e ^other types include sequence tagged allelic restriction sites (31), erythrocyte antigens (10), single strand conformational polymorphisms (6), serum protein markers (5), phenotypic observations (2) and sequence tagged site (2) markers from previous bovine maps[4,5,12].

A total of 918 SNP were identified in this study; 799 from EST sequences [GenBank Accessions BV103715 to BV106354] and 119 from BAC subclone sequence [Genbank Accessions BV445418 to BV446557; [13]]. One or both of the half-sib *Bos taurus *(Gelbvieh × Simmental) sires are heterozygous for 46% (380/834) of the SNPs genotyped in the two sires, and one or both of the *Bos taurus *× *Bos indicus *(Brahman × Angus; Nelore × Hereford) sires were heterozygous for 78% (706/908) of the SNPs genotyped (Additional file 2). Given costs of sequencing and genotyping, not every sire was sequenced or genotyped for every SNP. Because of an early observation that SNP were more prevalent in the *Bos taurus × Bos indicus *sires, there was a tendency to examine those bulls first, so the *Bos taurus *sires, progeny and mates may or may not have been genotyped for SNPs detected in *Bos indicus *cross animals.

Eighty percent of the SNPs developed in this study (Additional file 2) were positioned on the linkage maps. The 183 unmapped SNPs include 47 that did not have significant two-point linkage (lod > 3.0) to markers on the 1997 MARC linkage map [4], and 136 that were assigned to linkage groups but not placed on the multipoint map because they increased length of the linkage group excessively. A similar percentage of attempted SNP were placed on the multipoint swine linkage maps swine using similar genotyping and map construction strategies (G. Rohrer, personal communication). Unidentified genotyping errors are one possible cause of failure to map markers, both failure to detect significant twopoint linkage and rejecting markers inflating the map. Re-genotyping and verifying genotypes by sequencing, however, did not reveal systematic errors with the genotyping system. A more probable cause of individual marker failure may be the limited ability of the software to solve positions for biallelic markers with a small number of informative meioses encompassing a high percentage of like-heterozygote parents and offspring. Maps will inflate if a marker is placed incorrectly relative to other markers on the map, and if the most likely placement solvable by CRIMAP [14] results in an inflated map, that marker is rejected rather than allowing it to remain on a potentially distorted map. More correct placements may not be solvable by CRIMAP on 32-bit processors because the computations require more memory than can be addressed by 32-bit processors.

**Figure 1:**
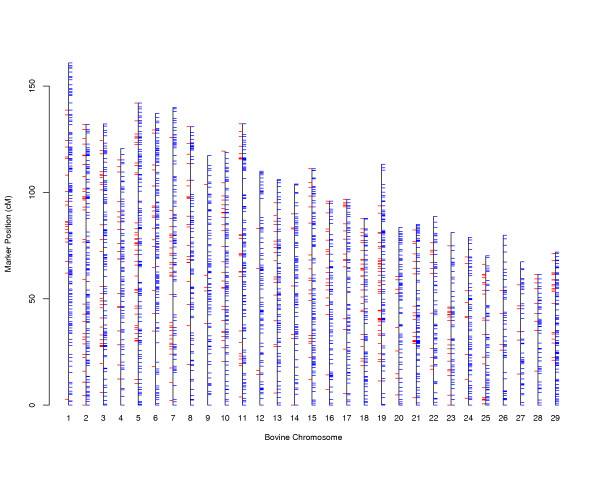
**Marker positions on bovine autosomes. **Marker positions on bovine autosomes. Vertical lines represent an individual chromosome. Red ticks to the left of each vertical line represent positions occupied by one or more newly developed SNP markers. Blue ticks to the right of each vertical line indicate positions occupied by other markers.

The 4585 autosome markers (Table 1) are placed in 2475 unique positions, with a mean (maximum) of 1.2 cM (9.1 cM) between markers. This is only slightly more dense than the maps without these SNPs [5], which contained 3755 markers in 2306 positions covering 3013 cM on autosomes (1.3 cM spacing). The new SNP markers occupy 511 distinct positions, 176 positions only represent these SNP and 335 are shared with other markers. The mean interval between the new SNP markers is 4.7 cM, with intervals up to 59.3 cM (Figure 1; Table 2). Each autosome contains at least one gap of 8.5 or more cM between SNP positions. SNP markers are spaced evenly along some chromosomes, other chromosomes contain clusters of several SNPs within a few cM. (The coefficients of variation in Table 2 provide relative measures of marker spacing; low values are indicative of even spacing, high values indicate uneven spacing with clusters of close markers separated by gaps.) Total length of the autosomes is 3058 cM, 45 cM longer than the 2004 microsatellite map [5], and 294 cM longer than the 1997 map [4]. The increased length, relative to the 2004 map, is due to recombinations introduced by new marker genotypes. These apparent recombinations may largely be attributed to incorrect ordering, when possibly more likely orders could not be solved by CRIMAP [14].

**Table 2:** Distribution of markers placed on bovine autosomes.

	**Markers^a^**		**Positions^b^**		**Human Connections^c^**		**Mean Interval^d^**		**Maximum Interval^e^**		**Marker Spacing CV^f^**	
	
**BTA**	**New SNP^g^**	**Other^h^**	**New SNP**	**Other**	**New SNP**	**Other**	**New SNP**	**Other**	**New SNP**	**Other**	**New SNP**	**Other**
1	28	263	21	150	18	30	6.8	1.1	59.3	5.7	1.9	.9
2	39	188	31	112	33	34	4.4	1.2	17.5	6.3	.9	.9
3	45	174	29	99	37	25	4.2	1.3	16.5	5.4	1.1	.8
4	28	131	18	82	21	21	6.1	1.5	16.2	4.6	.7	.7
5	62	185	36	114	40	37	3.8	1.3	22.8	4.7	1.3	.8
6	15	230	14	115	7	35	8.6	1.2	16.7	9.1	.6	1.0
7	45	142	28	88	39	31	4.6	1.6	34.3	8.5	1.5	.9
8	24	125	21	85	19	11	5.6	1.6	18.5	7.9	.9	1.0
9	5	126	5	79	5	10	16.3	1.5	42.7	4.9	1.1	.8
10	39	131	24	87	34	25	3.7	1.4	8.6	3.8	.7	.7
11	28	199	22	106	23	28	5.4	1.3	35.5	7.6	1.6	1.0
12	4	122	4	79	3	10	22.9	1.4	47.9	8.9	1.0	.9
13	26	116	17	75	21	17	3.9	1.4	12.1	4.9	.9	.7
14	6	133	6	77	6	21	18.0	1.4	49.5	5.1	1.2	.9
15	28	145	19	92	27	38	5.5	1.2	16.4	5.6	.9	.9
16	27	101	20	66	19	6	4.7	1.5	28.5	5.2	1.3	.7
17	23	107	15	70	16	15	6.5	1.4	24.4	5.5	1.1	.7
18	49	114	27	69	39	20	2.7	1.3	10.8	5.3	.9	.8
19	39	166	32	94	32	81	2.4	1.2	12.7	6.7	1.2	1.1
20	11	119	8	64	8	12	6.8	1.3	16.1	5.2	.8	.8
21	20	123	16	71	19	15	4.8	1.2	26.3	4.0	1.5	.7
22	25	84	12	57	20	21	5.4	1.6	18.7	7.2	1.1	.8
23	29	84	16	46	25	29	5.0	1.8	30.8	6.3	1.6	.7
24	10	95	8	62	7	9	9.5	1.3	17.3	3.8	.6	.6
25	29	74	25	51	21	12	2.7	1.4	11.3	7.1	1.3	1.1
26	12	64	4	44	11	8	9.4	1.9	14.5	7.2	.6	.9
27	5	72	5	41	1	6	10.5	1.7	15.9	6.2	.5	.8
28	9	79	9	44	7	10	5.7	1.4	19.0	4.7	1.0	.7
29	25	158	19	80	24	27	2.8	.9	13.6	3.3	1.2	.8

^a^Number of markers.

^b ^Number of distinct positions.

^c ^Number of markers with homologous human sequence, determined by BLAT alignments between marker sequences and UCSC hg17 sequence..

^d ^Mean interval between distinct positions.

^e ^Maximum interval between distinct positions.

^f ^Coefficient of variation for marker spacing. Low values indicate relatively even space between markers; high values indicate uneven spacing with clusters of markers separated by gaps.

^g ^Newly developed and mapped EST- and BAC-based SNP.

^h ^All other markers, including markers from previously described maps and newly mapped markers with data contributed by other projects.

Adding SNP markers resulted in minimal rearrangement of the linkage map. Correlations between marker positions on the 2004 [5] and current maps were near unity (r > .99) for all autosomes. Eighty-nine percent of the markers on autosomes from the 2004 map remained in the same index position (markers indexed 0,1,2 ... n on each autosome). Eight percent were shifted by a single position, and four percent moved by two to five positions. The most substantial shifts were with ambiguously placed markers, which can be included at any one of several positions without affecting map likelihood.

Adding EST- and BAC-based SNPs substantially increased comparative points defined by alignment of marker sequences against the human genome. Human homologues were identified for 1159 markers, including 498 markers from the 2004 map [5]. Newly developed SNP add 582 homologies, and 79 are from other markers added to the multipoint maps.

Utility of the current map to explore regions of the genome that influence animal performance can be demonstrated by examining the regions surrounding previously described QTL. A region of BTA 5, from 73.5 to 77.6 cM on the 1997 MARC map [4], bounded by microsatellite markers IGF-1 and BM1819, has been associated with preweaning gain [15]. Allowing for changes in the map, the region defined by these two markers now spans 76.9 to 84.6 cM. The two markers are separated by one marker on the 1997 map. The 2004 SIAG-MARC map [5] placed nine microsatellites in the region; and the current map shows the same nine microsatellites as well as five SNPs. The recent bovine RH map [6] contains four gene markers and the BM1819 microsatellite in the corresponding region, from 326.8 to 337.8 cR_5000_. Three of the gene markers (IGF1, TU12B1-TY, SYCP3) were aligned with human chromosome (HSA) 12 sequence, from 100.6 to 102.7 Mbp. The fourth gene marker (TIMP3) aligned with HSA 22 at 31.5 Mbp. Sequences associated with linkage markers also align with HSA 12 and 22. Sequence associated with microsatellite DIK4787 aligns with HSA 12 at 105.2 Mbp. Sequences containing an SNP (17019_98) and two microsatellites (BMS1216, DIK5165) align with HSA 22, from 31.2 to 32.0 Mbp. These alignments suggest that the QTL region contains a break in synteny, in agreement with the break described by the bovine RH – human comparative map [6].

Agreement in comparative alignments between the current linkage and RH maps suggests either map might be used to identify positional candidate genes underlying this QTL. Taken together, information from both maps may provide more complete coverage than that indicated by either map alone. The polymorphic linkage markers will be more useful to identify relationships between marker genotype and phenotype and fine-map the QTL, although further marker development will be necessary to isolate causative polymorphisms.

Accuracy of placing SNPs on the linkage map was a concern, because bi-allelic SNPs are generally less informative than highly polymorphic microsatellite markers (Table 1). Most markers (80%) with three or more alleles have unambiguous placement, while a single, most likely, position can be determined for only 36% of the bi-allelic markers, and the mean 3.6 cM three-lod confidence interval for multi-allelic markers is narrower than the 8.5 cM confidence interval for bi-allelic markers.

## Discussion

The current linkage map, representing over 4000 anonymous markers and several hundred gene-specific SNPs, provides a resource to link genetic variation in animal performance to underlying DNA sequence variation. The procedures used to construct this map were designed to allow frequent updates, so new markers can easily be added. The reduction of mean space between markers, from 3.0 cM to 1.3 cM, primarily because of recently added microsatellites [5], increases opportunities to fine-map QTL. Addition of EST-based SNPs increases connections between the genetic map and gene maps, including currently available bovine RH maps, annotated human and model organism sequence, and eventually bovine sequence [16]. These connections may increase efficiency of identifying genes and causal mutations affecting animal performance. Where QTL regions can be narrowed, and the regions include markers connecting the region to annotated sequence, the list of positional candidate genes that might partially explain phenotypic variation may be shortened considerably.

The current or future versions of the bovine genetic map will be useful to assemble and validate bovine genomic sequence. The genetic map represents the intact living genome, so it is not subject to cloning and assembly problems associated with other mapping and sequencing techniques [17]. However, resolution of the genetic map is limited, and markers that were not separated by recombination in the experimental population cannot be correctly ordered on the linkage map. Genetic mapping data can be combined with bovine RH [6,18] and BAC map data [19], exploiting resolution characteristics of both genetic and physical mapping data [20,21] to obtain a high resolution, well ordered consensus map useful to guide and refine bovine sequence assembly [17,22], and anchor QTL on the draft sequence [23]. Even after complete assembly of the bovine genome, the sequence will not replace the genetic map. The genetic map, especially if it is continually updated to represent new SNPs and other markers, should provide valuable links between phenotypes and associated marker genotypes, in order to identify and exploit genomic variation influencing economically important traits.

## Conclusion

More than 700 EST- and BAC-based SNP markers were added to the bovine linkage map. Order of previously mapped markers was largely unaffected. The SNPs increased the density of the map somewhat, and substantially increased connections to gene-rich physical maps, including annotated human sequence. The number of linkage markers with human homologues was more than doubled by addition of these SNP and other markers. The map provides a resource for fine-mapping quantitative trait loci and identification of positional candidate genes, and can be integrated with other data to guide and refine assembly of bovine genome sequence. The map can easily be updated with a cyclic map construction process, and it will continue to be a useful resource connecting observable phenotypes and animal genotypes to underlying genes and molecular mechanisms influencing economically important beef and dairy traits.

## Methods

### Marker development

Single nucleotide polymorphism markers were developed from bovine EST sequence as described [11]. Briefly, tentative consensus (TC) clusters of bovine EST were obtained from The Institute for Genomic Research (TIGR) Bovine Gene Index[24]. Repeat elements in the TC were masked using RepeatMasker [25] and aligned with the human genome draft sequence via BLASTN [26]. Alignments were checked for the presence of apparent introns using a perl script that computed the predicted intron size. Primers were designed from the bovine EST sequence in such a way as to cross introns and produce products of approximately 800–1300 bp, while including at least 100 bp of putative exon sequence to allow confirmation that the primers targeted the desired gene.

Alternatively, SNP for some genes were developed from sequence associated with bacterial artificial chromosome (BAC) clones carrying all or part of the target gene(s) essentially as described [27]. Briefly, high density filters representing the CHORI-240 BAC library (P. deJong, personal communication) were screened with radiolabeled insert from EST clones representing target genes as recommended by the manufacturer (BACPAC resources, Oakland, CA). Positive clones were partially digested using the restriction enzyme Sau3AI (Promega, Madison, WI) by incubation of isolated BAC DNA. Aliquots of the reaction were removed at 10, 20 and 30 minutes of incubation into 10 mM final concentration EDTA on ice, desalted by gel filtration column (Axygen, Union City, CA), and separated on a 0.8% agarose gel. Fragments of 0.8–1.5 kilobase (kb) were excised from the gel and purified by ion exchange column as directed by the manufacturer (Marligen, Ijamsville, MD). Isolated fragments were cloned into pBluescript vector (Clonetech, Palo Alto, CA) prepared by digestion with restriction enzyme BamHI, and 192 randomly selected subclones for each BAC were picked into 80 ul LB media supplemented with ampicillin at 50 ug/ml in 384-well plates for sequencing from both ends with vector primers. A total of 134 BACs were screened, representing 122 loci (approximately 0.3% of the bovine genome).

Resulting sequences were analyzed using Phred, Phrap, and Consed programs [28] and amplification primers were designed using the autofinish and primer3 options of Consed. Primers derived from EST sequence or BAC subclones were used to amplify DNA from four bulls that were the sires (two *Bos taurus × Bos taurus*; two *Bos taurus *× *Bos indicus*) of the MARC reference panel mapping families [12], and the PCR products were sequenced with the amplification primers to identify heterozygous positions (SNP) in the amplicons using Polyphred and Consed. The SNPs were genotyped in progeny and mates of heterozygous sires from the MARC reference population using the Sequenom MassArray System [29] following established procedures [11].

### Map construction

#### Software and procedures

Map construction was an iterative process completed in cycles. Cycles were initiated when genotypes for new markers, or corrections to previously genotyped markers, were recorded for animals in the MARC reference population. CRIMAP 2.4 [14], modified to reduce occurrence of unsolvable marker orders and controlled by a series of Perl scripts, was used to assign markers to linkage groups, order markers in each linkage group, and identify possible genotyping errors. All markers genotyped in the reference population were considered in map construction, including SNPs developed for this project as well as microsatellites, SNPs and other types of markers with recorded genotypes.

The modifications to CRIMAP included redimensioning arrays to accommodate a larger number of markers, and using logarithmic arithmetic for intermediate calculations to avoid values exceeding the precision limits of the 32-bit CPUs used to solve the maps. The perl scripts were developed to construct sets of alternative marker orders necessary at each step, distribute the needed CRIMAP *fixed *executions to nodes of a Linux cluster, and identify the most likely order from the set of orders evaluated in each step.

#### Mapping data sets

Two data sets were used to construct maps. Final marker order and map distances were computed from the complete reference population pedigree and genotype data. A reduced data set was used to initially place new markers and order the maps. The reduced data set was constructed by eliminating progeny genotypes where the progeny and both parents had identical heterozygous genotypes. These ambiguous, like-heterozygous, genotypes provided little information about recombination, although phase of inheritance and recombination are inferred from linked markers with unambiguous genotypes. Genotypes for about one-half of the markers included at least one ambiguous genotype, but fewer than 2.5% of the total number of observed genotypes were eliminated from the reduced data set. Including these ambiguous genotypes increased the number of calculations and computer memory necessary to compute likelihood of a particular marker order. Likelihoods of certain marker orders, usually involving several markers with ambiguous genotypes ordered consecutively, could not be solved using the complete data set. When the reduced data set was employed, uncomputable orders were eliminated and the time required to solve individual likelihoods reduced, making it feasible to evaluate a larger number of alternative marker orders.

#### Linkage group assignments

A map construction cycle began by extracting genotypes and pedigree data from the MARC database, and formatting the full and reduced data sets. Genotypes exhibiting non-Mendelian inheritance patterns were detected by the CRIMAP *prepare *option. Two-point analyses, with the complete data, were conducted to assign newly genotyped markers to linkage groups representing entire chromosomes. The two-point analyses established linkage between the new markers and subset of markers from the 1997 MARC map [4], selected to contain the most informative marker within each 5 cM interval. A LOD score greater than 3.0 was required to assign a new marker to a linkage group. Markers were assigned to the linkage group containing the marker with the highest two-point LOD score with the lowest recombination fraction.

#### Initial ordering of markers within linkage group

Markers assigned to a linkage group were initially ordered using the reduced data set. Starting from the existing order of a linkage group, each marker assigned to that linkage group by two-point analysis, but not present on the multi-point map, was tested in every possible location. These unmapped markers were evaluated in order of decreasing informativeness; the marker with the greatest number of informative meioses was tested first, and the marker with the least informative meioses was the last evaluated in each round of marker insertion. Markers were inserted at the location with the highest log-likelihood, if placement at that position did not increase map length by more than 0.75 cM. This somewhat arbitrary limit on increases in map length was imposed to minimize distortion that can result from errors in genotypes and marker order. Once a set of markers meeting the allowable increase in map length was inserted, alternative marker orders were evaluated in an iterative procedure until a more likely order could not be identified. Log-likelihoods were computed with each pair of adjacent markers interchanged. The current order was then replaced by the order of the switched pair showing the greatest improvement in likelihood, and the process was repeated until switching pairs of adjacent markers did not improve likelihood of the map. Map lengths with each marker temporarily removed from the linkage group were then determined. If removing a marker reduced map length, that marker was evaluated in all possible positions and reinserted at the position with the highest likelihood. If order was changed by removing and reinserting markers into more likely positions, the reordering process, starting with interchanging adjacent pairs of markers was repeated. If the resulting marker order was different than the initial order from the previous attempt to insert markers, and some markers assigned to the linkage group remained unmapped, processing continued with another attempt to insert markers.

#### Finalizing maps

After no more markers could be inserted that satisfied the criteria defined above, and the algorithms did not reveal a more likely marker order from the reduced data, the full data set was used to finalize the set of markers on the multi-point map, then order and position those markers. Inferred inheritance of ambiguous genotypes, included in the complete data set, suggested some map inflation and marker rearrangements that were not apparent in the reduced data set. Lengths of the map with markers individually removed were determined, and interior markers that stretched the map more than 0.75 cM were eliminated from the set of mapped markers. At this stage, the limit prevented map inflation caused by placing markers in the most likely solvable order, when possibly more likely orders, that did not increase map length, could not be solved using the complete data. The process of switching adjacent marker pairs was repeated with the full data set to establish a final marker order, and marker positions were computed from this order. The final maps represent the most likely marker order identified with the complete data set, although an exhaustive search of all possible marker orders was not conducted (and is not feasible).

After determining the final marker order, the *chrompic *option of CRIMAP was used to determine likely grandparental origin of marker alleles and identify recombination along each progenys' chromosomes. Several *chrompic *analyses were conducted for each linkage group; one for the final map and one for each marker that increased map length by more than 0.75 cM. Possible genotyping errors, indicated by two or more recombinations in an individual's chromosomes, were identified and suspicious genotypes were checked by manual inspection of the raw spectrographic data. Where no apparent error in the assay could be detected, animals were genotyped a second time using the Sequenom system, and in selected cases, genotypes were verified by sequencing. Corrections were entered in the database, and used in subsequent map construction cycles.

#### Confidence interval estimation

Confidence intervals around marker positions were estimated by computing the likelihoods of each marker in all possible positions, while preserving the final order of remaining markers in the linkage group. These likelihoods were computed with CarthaGene [20] using output translated from the CRIMAP [14]*chrompic *analysis of the final marker order. CarthaGene was used primarily for speed; the CarthaGene analyses ignored the distinction between probable and known allele phase, but yielded similar results in substantially less time and eliminated any occurrence of uncomputable orders. For a given LOD threshold, alternate positions yielding a log-likelihood difference less than the threshold were determined, as well as map positions (cM) of markers holding that order. Confidence intervals were computed from map positions corresponding to marker order. A map containing *n *markers ordered *1, 2, ... n *had corresponding positions *p_1_, p_2_, ... p_n_*. An individual marker *m *holding order *i *could be placed anywhere between the position corresponding to the left index *li *to the position corresponding to the right index *ri *(*li <= i <= ri*) without reducing log-likelihood more than some threshold *t*. Confidence intervals were estimated from the equations:

*CI*_*mt *_= *p*_*li *+ *1 *_- *p*_*ri *- *1 *_for *li *> *1 *and *ri *<*n*

*CI*_*mt *_= *p*_*li *_- *p*_*ri *- *1 *_for *li *> *1 *and *ri *= *n*

*CI*_*mt *_= p_*li *+ *1 *_- p_*ri *_for *li *= *1 *and *ri *<*n*.

The estimated confidence intervals are bounded by 0 and p_n_, so intervals including either end of the linkage group may be underestimated.

### Comparative mapping

The collection of bovine sequences in GenBank sequence tag site (STS), mammal (MAM), patent (PAT), EST, and genome survey sequence (GSS) divisions, excluding sequences from the ongoing bovine sequencing effort [16], were obtained. Bovine sequences associated with markers were identified with e-PCR [30]. Where the primer pair for a marker matched multiple sequences, a consensus sequence representing that marker was obtained with phrap[28], and repetitive sequence was masked [25]. BLAT [31] was used to align resulting sequences to the May 2004 human genome assembly [32]. The highest scoring alignment for each marker sequence was identified, and was considered comparative only if all high scoring alignments for that marker consistently aligned with the same region of the human genome. Marker-human alignments were discarded if the marker sequence aligned with two or more regions of the human genome with similarly high BLAT scores.

## Authors' contributions

WMS developed map construction procedures, conducted linkage analyses and drafted a major portion of the manuscript. EC organized DNA sample collection and storage. RTS and TPLS developed SNP markers and genotyped animals. JWK developed procedures to record and manage genotypes. GPH conducted sequence analyses, including masking repeats in EST sequence, BLAST alignments, and primer design. GLB oversees linkage data collection and curates the maps. All authors were involved in conceiving and planning the research, which was coordinated by TPLS. All authors read and approved the final manuscript.

## Supplementary Material

Additional File 1 Excel spreadsheet containing linkage maps of bovine autosomes. Data include marker name, locus, and type; PubMed or other reference, chromosome, position on chromosome, 3-lod confidence interval and comparative position on human sequence. Click here for file

Additional File 2 Excel spreadsheet containing details about each newly developed SNP. Data include MARC id number, marker name, GenBank STS id, GenBank Accession, primer sequneces, and results from twopoint and multipoint linkage analyses. Click here for file
